# Piezo1 activation facilitates ovarian cancer metastasis via Hippo/YAP signaling axis

**DOI:** 10.1080/19336950.2022.2099381

**Published:** 2022-08-08

**Authors:** Yanjie Xiong, Liru Dong, Yun Bai, Hui Tang, Shuang Li, Dan Luo, Fang Liu, Jie Bai, Shikun Yang, Xudong Song

**Affiliations:** a Department of Pathology, North China University of Science and Technology Affiliated Hospital, Tangshan, Hebei China; b College of Life Science, North China University of Science and Technology, Tangshan, Hebei China

**Keywords:** Piezo1, ovarian cancer, tumor mechanical environment, EMT, Hippo/YAP signaling

## Abstract

Ovarian cancer (OC) is a highly malignant cancer with great metastatic potential. Here we aimed to investigate the role of Piezo1, a gene related to the mechanical environment of the tumor, in promoting the metastasis of OC. We performed Piezo1 knockdown in A-1847 cells using small hairpin RNAs, and the cells were inoculated subcutaneously in nude mice. Piezo1 knockdown decreased the tumor growth rate of OC tumor xenografts in mice and reduced cell migration *in vitro*. Metastasis in the lung was also attenuated after Piezo1 knockdown as revealed by HE staining of the lung tissues, which was concomitant with downregulation of E-Cadherin and vimentin and upregulation of N-Cadherin analyzed using western blot analysis, suggesting suppressed epithelial-to-mesenchymal transition. Migration of Piezo1-knockdown cells was also analyzed for their migratory capabilities using the scratch assay. We also analyzed the key proteins in the Hippo/YAP signaling pathway using western blot after treating A-1847 and 3AO cells with a Piezo1 inducer, Yoda1. Piezo1 inducer Yoda1 activated Hippo/YAP signal in OC cells. In conclusion, Piezo1 is overexpressed in OC tissues and contributes to OC tumor growth and metastasis. Suppression of Piezo1 is a potential therapeutic strategy for OC.

## Introduction

Ovarian cancer (OC) is a highly malignant tumor originated from the ovary, and the morbidity and mortality of OC are on the rise in recent years [1]. At the time of diagnosis, many OC patients show local infiltration or distant organ metastases, thereby becoming ineligible for tumor surgical resection [2]. Treatment options are also limited by the lack of understanding about how the agranulocytes metastasize. Therefore, elucidation of the molecular underpinnings of the metastatic cascade before OC metastasis is important.

Metastasis is a major cause of cancer-related mortality. It has been demonstrated that cancer cell motility is regulated by the mechanical environment of cancers [3]. Interestingly, recent studies have underscored increased interstitial fluid pressure as a driving force for metastasis [4]. Piezo-type mechanosensitive ion channel component 1 (Piezo1) has a widespread expression in non-sensory cells and serves as one of the key sensors of mechanical stimuli [5–7]. Piezo1 is involved in sensing the shear stress erythrocytes and vascular endothelial cells were exposed to, and participates in regulating urine flow perception [8]. In addition, recent reports have associated aberrant Piezo1 with poor prognosis in certain types of cancer [1,9]. It is however worth noting that the role of Piezo1 in cancer progression appears to be tissue-dependent. Piezo1 overexpression has been observed in several cancers, including bladder cancer [10], breast cancer [1], gastric cancers [6] and osteosarcoma [11]. In line with this, Piezo1 knockdown inhibits the adhesion capacity of these cancer cells, thereby preventing cell metastasis. On the contrary, Piezo1 knockdown was found to accelerate lung cancer progression [12]. To date, the role of Piezo1 in OC has yet to be clarified.

Herein, we aim to determine whether Piezo1 is involved in OC development, focusing on whether Piezo1 is associated with OC tumorigenesis and capable of driving OC metastasis. We further explore the possible molecular mechanisms of Piezo1 in OC by focusing on the Hippo/YAP pathway, which has been shown to be associated with abnormal tissue growth and tumorigenesis of multiple cancers [13], including OC [14]. Our study could clarify the role of Piezo1 in promoting OC development and pave the way for translating Piezo1 as a diagnostic and therapeutic target in OC to clinics.

## Materials and methods

### Tissue collection

Our experiments were approved by the institutional review board of North China University of Science and Technology Affiliated Hospital. Human primary OC tissues and adjacent non-tumor tissues were collected. Written informed consent was derived from all participants. Tissue samples were snap-frozen and kept at −80°C until use.

### Quantitative RT-PCR

Total RNA from primary OC tissue, non-tumor tissue samples or cells was extracted using the TRIzol reagent (ThermoFisher Scientific, Waltham, MA). The RNAs of 1 μg were then reversely transcribed into cDNAs using the SuperScript cDNA synthesis kit (ThermoFisher Scientific). The Eppendorf mastercycler realplex system and SYBR Green Mix were used for RT-PCR using Mastercycler Mastercycler nexus (Eppendorf, Hamburg, Germany) under the following conditions: Each cycle consists of denaturation at 94°C for 1 min, annealing at 55°C for 2 min, and elongation at 72°C for 2 min and 30 cycles were conducted. The final cycle was followed by extension at 72°C for 5 min. The primers used in the study included: *Piezo1*: forward: CCTGGAGAAGACTGACGGCTAC, reverse: ATGCTCCTTGGATGGTGAGTCC. *GAPDH*: forward: GTCTCCTCTGACTTCAACAGCG, reverse: ACCACCCTGTTGCTGTAGCCAA. *GAPDH* was used as the house-keeper gene.

### Western blot analysis

Proteins were extracted from tissue samples by lysis in the RIPA buffer, followed by quantification using the BCA assay (Abcam, Cambridge, MA). Six pairs of human OC and non-tumor tissue samples from six cases were used for western blot analysis of Piezo1 protein expression. Protein lysates of 10 μg was loaded to 10–12% gels for sodium dodecyl sulfate–polyacrylamide gel electrophoresis and then electro-transferred to nitro cellulose membranes. 1% BSA in TBS-Tween 20 (0.1%) was used to block nonspecific binding. The rabbit primary antibody specific to Piezo1 (cat. # ab128245, 1:1000 dilution, Abcam) was then added to incubate the membrane overnight at 4°C. After phosphate-buffered saline washing, mouse anti-rabbit IgG HRP-conjugated secondary antibodies were then added (1:500 dilution) and incubated the membrane for 1 h at room temperature. All other antibodies specific to Hippo/YAP signaling pathway were purchased from Cell Signaling (Danvers, MA). To visualize protein bands, ECL substrates were added and a ChemiDoc imager (Biorad, Hercules, CA) was used to acquire the images of the membrane.

### Cell culture and scratch assay

The human OC cell lines, A-1847 and 3A0 were acquired from ATCC (Manassas, VA). RPMI medium supplemented with 10% fetal bovine serum was used to culture the cells at 37°C and 5% CO_2_. For scratch assay, cells were cultured on six-well plates to 89–90% confluence, and a straight scratch was made using a sterile 10 μL pipette tip. After 24 h, the width of the scratch was measured on images acquired under a light microscope.

### Transfection of shRNAs

The plasmid containing shRNA specific to Piezo1 (Origene, Cat # TF313084) or the Veh-shRNA (Origene, cat # TR20003) was transfected to A-1847 cells cultured in 6-well plates in the serum-free medium using lipofectamine 2000 (ThermoFisher Scientific).

### Animal studies

The Balbc nude mice (female, 8–12 weeks) were acquired from Cyagen Biosciences Inc. (Suzhou, China). Two million A1847 cells with Piezo1-shRNA or Veh-shRNA transfection were subcutaneously injected into the flank of mice. The growth of the tumor was measured weekly until 8 weeks. The tumor volume was calculated as length × width [2]/2. At the end of 8 weeks, tumors were collected after sacrificing the mice.

### Statistical analysis

All data were acquired based on at least three independent measurement and expressed as mean ± SD. The student’s t-test was used to compare two groups. One-way ANOVA with a Tukey post hoc test, and two-way ANOVA analysis with a Bonferroni post hoc test were used to compare multiple groups. The differences with p < 0.05 were considered statistically significant.

## Results

### Piezo1 is upregulated in ovarian cancer patients

**Figure 1.**
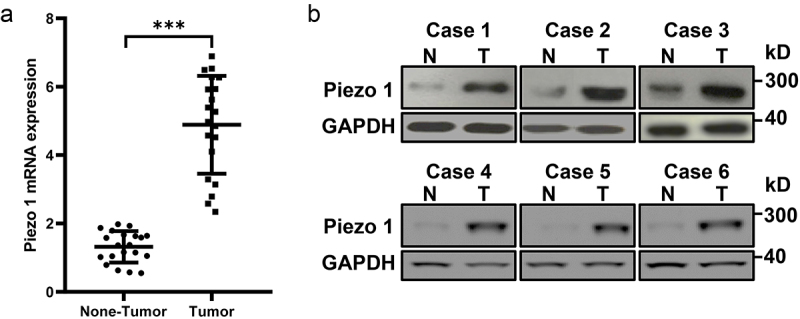
**Piezo1 is upregulated in human OC tissues**. (a) Analyses of Piezo1 mRNA expression, and (b) protein expression by Western blot in human primary OC tissues and adjacent non-tumor tissues. Data were expressed as mean ± SD. ***p < 0.001.

We evaluated Piezo1 in tumor tissues and adjacent tissues of OC patients by RT-PCR, western blot, and immunohistochemical analysis. As expected, Piezo1 mRNA was shown to be significantly higher in tumor tissues than non-tumor tissues (n = 20, Figure 1a). Also, Piezo1 expression was higher in ovarian cancer tumor tissues than that in adjacent non-tumor tissues (Figure 1b). The quantitative analysis of Piezo1 expression in the protein level is shown in **Figure S1**.

**Figure 2.**
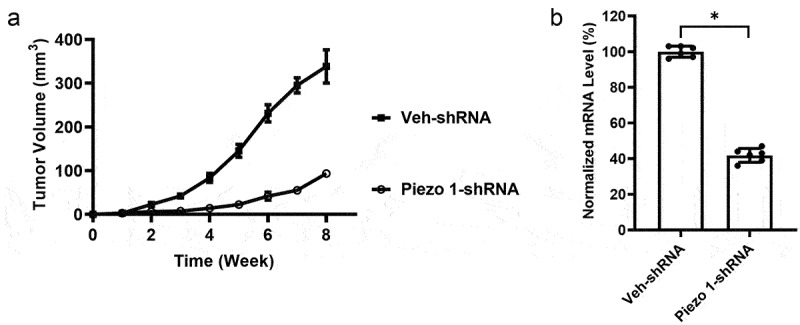
**Piezo1 knockdown inhibited OC growth in vivo**. (a) Tumor volume of A-1847 xenografts with or without Piezo1 knockdown. n = 6 for each group. (b) Piezo1 mRNA levels of tumor collected from different groups. Data were expressed as mean ± SD. *p < 0.05.

To evaluate how Piezo1 affects tumor growth, A-1847 cells transfected with Veh-shRNA or Piezo1-shRNA were used to initiate tumor xenografts in nude mice. As shown in Figure 2a, tumors grew slower and smaller after *in vivo* inoculation of Piezo1-shRNA-transfected A-1847 cells compared to mice inoculated with Veh-shRNA-transfected A-1847 cells (Figure 2a). qPCR data showed that, compared to transplanted tumors from A-1847 cells transfected with Veh-shRNA, A-1847 xenografts with stable Piezo1 knockdown had significantly lower expression of Piezo1 (Figure 2b).

**Figure 3.**
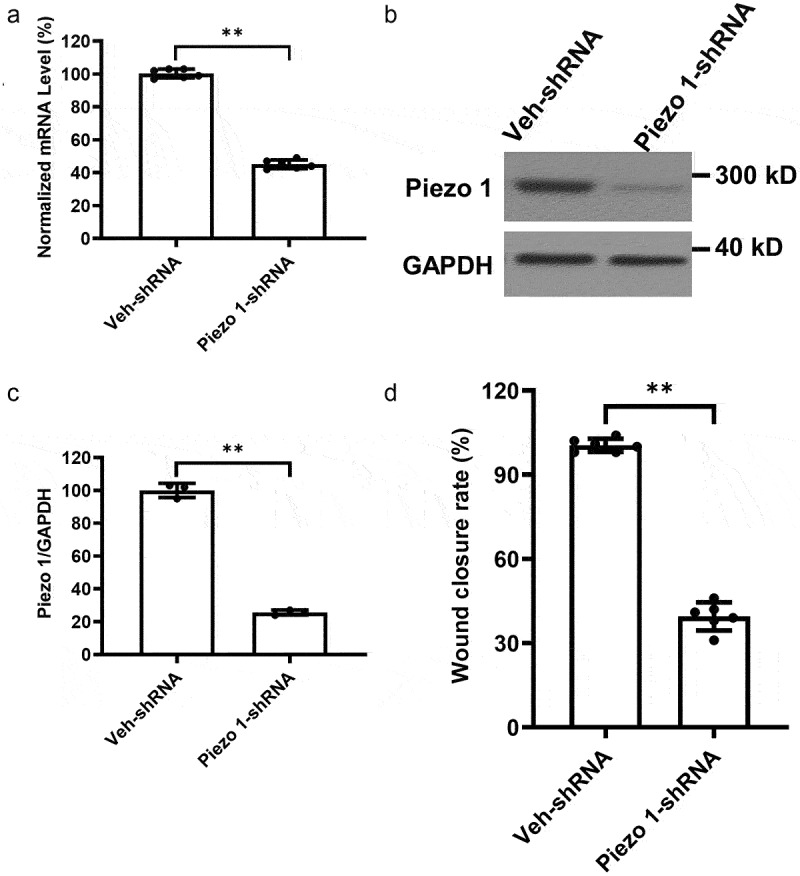
**Knockdown of Piezo1 gene expression suppressed cell migration in A-1847 cells**. (a) mRNA and (b-c) protein expression of Piezo1 in A-1847 cells after knockdown using Piezo1-shRNA. (d) Wound disclosure rate of A-1847 cells with or without Piezo1 knockdown. Data were expressed as mean ± SD. **p < 0.01.

We further investigated the effect of Piezo1 knockdown on the migration of A-1847 cells in vitro. As shown in Figure 3a–3c, mRNA and protein expression of Piezo1 were significantly reduced in A-1847 cells after transient transfection with Piezo1-shRNA. A-1847 cells with stable Piezo1 knockdown showed increased cell migration compared to those transfected with Veh-shRNA (Figure 3d).

### Ovarian cancer metastasis is suppressed by Piezo1 knockdown

**Figure 4.**
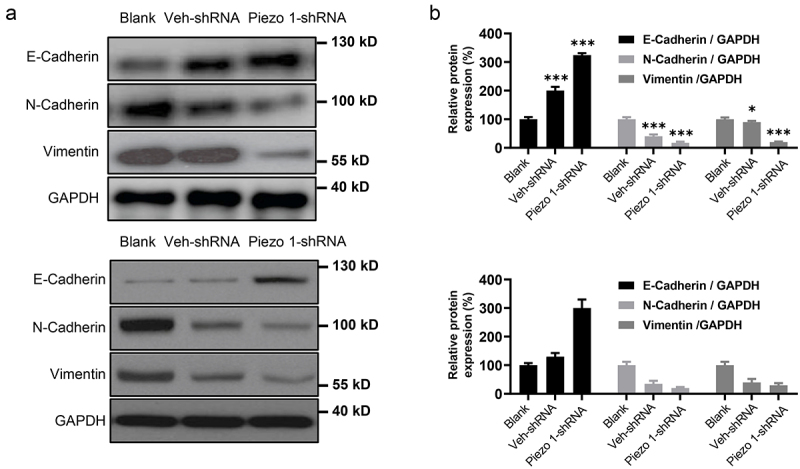
**Piezo1 promotes OC lung metastasis**. (a) Western blot and (b) Semi-quantitative analysis of protein expression of EMT-related molecules in metastatic nodules. Data were expressed as mean ± SD, n = 3. *p < 0.05, **p < 0.01, ***p < 0.001.

To verify the effect of Piezo1 knockdown on OC metastasis in vivo, A-1847 cells with Piezo1-shRNA or Veh-shRNA transfection were injected via tail vein. Western blot analysis suggested relatively higher expression of E-cadherin and lower expression of N-cadherin and vimentin in lung metastatic nodules of mice injected with cells with Piezo1 knockdown (Figure 4a–4b). Taken together, these data indicate that Piezo1 is an inducer of epithelial-to-mesenchymal transition (EMT) in OC cells and promotes metastasis in vivo.

### Piezo1 activation blocks Hippo signaling to activate YAP to promote OC metastasis

**Figure 5.**
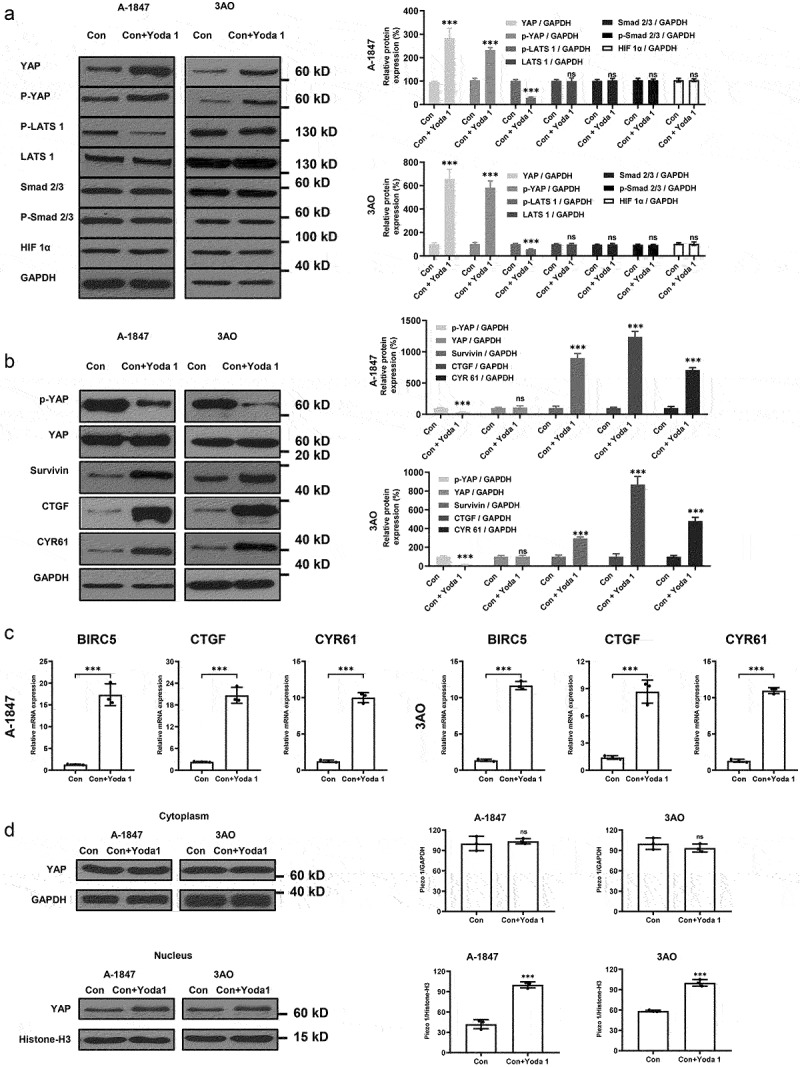
**Activation Piezo1 inhibits hippo/YAP signaling activation**. (a and b) Western blot and semi-quantitative analysis of protein expression. (c) mRNA levels of hippo signaling components measured by RT-PCR. (d) Western blot and semi-quantitative analysis of protein expression of YAP in the cytoplasm and nucleus. Data were expressed as mean ± SD. ***p < 0.001.

To further elucidate the molecular mechanism of Piezo1 in promoting EMT and metastasis of OC cells, we treated A-1847 and 3AO cells with the Piezo1 inducer Yoda 1. As a result, the expression of p-Smad 2/3, Smad 2/3, HIF-1a, and LATS 1 was not significantly altered by Yoda1 treatment, whereas activation of Piezo1 in OC cells did result in p-LATS1 downregulation and yes-associated protein 1 (YAP) upregulation. To our surprise, p-YAP (Ser127) was also increased, suggesting YAP activation (Figure 5a), which hypothetically can be attributed to substantial accumulation of total YAP. This hypothesis was tested by extracting equal amounts of YAP and measuring p-YAP expression. Expectedly, p-YAP was decreased by Yoda 1 treatment (Figure 5b). In addition, the expression of YAP target genes CTGF, BIRC5 (survivin), and CYR61 were upregulated (Figure 5c), further demonstrating that Yoda 1 treatment inhibited the Hippo signaling pathway. By analyzing nucleus proteins specifically and YAP target genes, our results suggested that YAP might be translocated to the nucleus and upregulate downstream genes following Piezo1 activation (Figure 5d). It is worth noting that Yoda1 is an important regulator of calcium circulation. Hence, we analyzed calcium flux through Fluo-8 calcium indicator in A1847 cells treated with 50 μM of Yoda1, and we found that Yoda1 increased intracellular calcium levels, which is crucial for the Piezo1-mediated cancer metastasis (**Figure S2**). Further, we show that in A1847 cells with Piezo1 knockdown, Yoda1 failed to promote cell migration. Whereas, in cells without Piezo1 knockdown, Yoda1 indeed promoted cell migration (**Figure S3**). The transwell assay (**Figure S4**) also suggested that Piezo1 knockdown promotes cell invasion. These evidences supported that Piezo1 is important for the metastatic ability of ovarian cells.

## Discussions

Here we found that significant upregulation of Piezo1, a sensor of the mechanical microenvironment of the tumor, was observed in tumor tissue compared to normal tissues, which was associated with a poor prognosis in patients with OC. Furthermore, mechanical force-induced Piezo1 activation was shown to block the hippo signaling pathway and increased OC cell metastasis.

Our findings are in agreement to the notion that cells can sense mechanical changes in the extracellular environment and initiate responses to maintain homeostasis by translating mechanical forces into cellular and biochemical responses. Indeed, it has been reported that OC cells are constantly stimulated by the high mechanical stress caused by obstruction [15], and our data clarified that this stress is associated with OC metastasis.

The current study suggests EMT, a driving force of OC development, is regulated by Piezo1, which provided a link between altered mechanical microenvironment to increased metastasis in agranulocytes. We found that Piezo1 was positively correlated with migratory capabilities of OC cells in vitro and lung metastasis in vivo, which is in agreement to the cancer-promoting role of Piezo1 as previously reported in studying gastric cancer, glioma, and osteosarcoma [6, 10–12]. Notably, a recent study also reported that Piezo1 deficiency accelerated the progression and cell migration of non-small cell lung cancer, suggesting that mechanical stimuli-mediated Piezo1 induction may also adopt an anti-cancer role [12]. This suggests that mechanical stimuli may lead to opposing cellular behaviors and our data is important to clarify the pro-cancer role of Piezo1 in OC development.

We also demonstrated that the Hippo/YAP signaling pathway is mediated by Piezo1, and therefore played an important role in Piezo1-mediated OC metastasis. By activating a series of kinase cascades, the Hippo signaling pathway is imminent in the physiological control of organ size [16]. Dysregulation of Hippo signaling has been shown to lead to abnormal tissue growth and tumorigenesis of multiple cancers [13], including OC [14]. The key proteins of the Hippo pathway include nuclear transcription factors (TEADs), upstream kinases (MST1/2 and LATS1/2), and the transcriptional co-activator YAP, the expression of which have been studied in the present study. Here, our data suggested that Piezo1 is a novel Hippo activator that facilitates LATS1 dephosphorylation and activates Hippo signaling. While the analysis of kinase activity was not performed in our study, it can be speculated that Piezo1 indirectly inhibits phosphorylation of LATS1, thereby exerting its cytoskeletal regulatory function. Alternatively, given recent studies showing that Hippo pathway in tumor may be affected by intracellular calcium levels [17,18], it is also possible that phosphorylation of LATS1 may be inhibited by intracellular calcium, which activates certain phosphatases. Further studies are needed to further explore these hypotheses.

Our study has several limitations. First, the biological behavior of the cells under mechanical stimuli was not analyzed in our study. Furthermore, our in vivo study did not simulate the mechanical environment of OC, which is needed to further verify the role of Piezo1 in OC with a biologically relevant mechanical environment.

## Conclusions

In conclusion, this study indicates that elevated Piezo1 expression is correlated with OC tumorigeneses. Mechanical stimuli induced the activation of Piezo1, which led to OC cell metastasis via the Hippo/YAP signaling. These results provide a basis for elucidating the mechanisms of OC development and support the possible use of Piezo1 as a diagnostic and therapeutic target in OC.

## Supplementary Material

Supplemental Material

## Data Availability

Data could be obtained upon reasonable request to the corresponding author.
