# Supplementary material for: Piezo1 activation facilitates ovarian cancer metastasis via Hippo/YAP signaling axis
Source: Channels (Austin). 2022 Aug 8;16(1):159–66. doi: 10.1080/19336950.2022.2099381 (PMC9367648; doi:10.1080/19336950.2022.2099381)
Supplement: Supplemental Material [file KCHL_A_2099381_SM2981.docx]

**Supplementary materials**


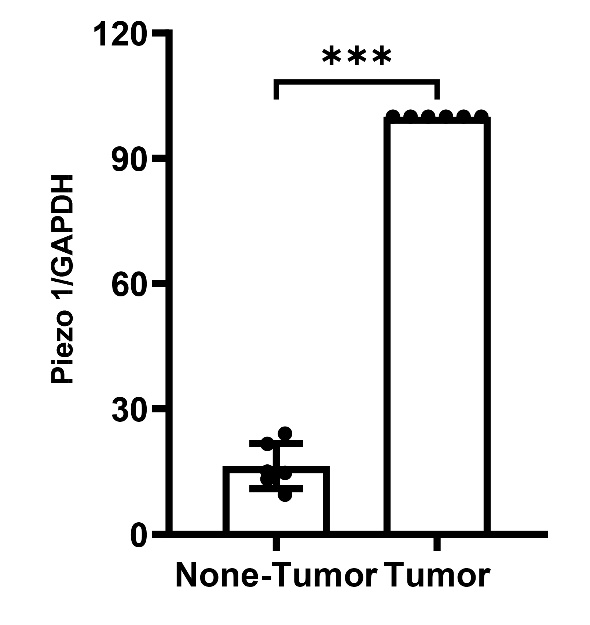


Figure S1. Semi-quantitative analysis immunoblot analyses of Piezo 1 protein expression in OC tissues and adjacent non-tumor tissues


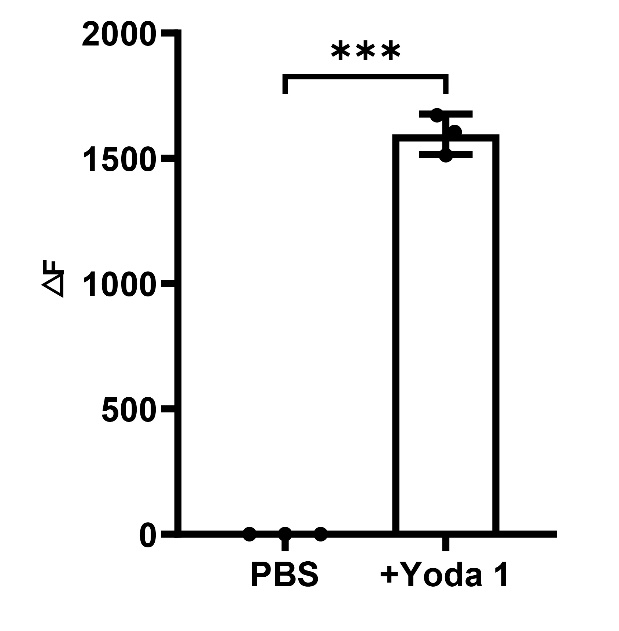


Figure S2. Yoda1-induced intracellular calcium signals in A1847.


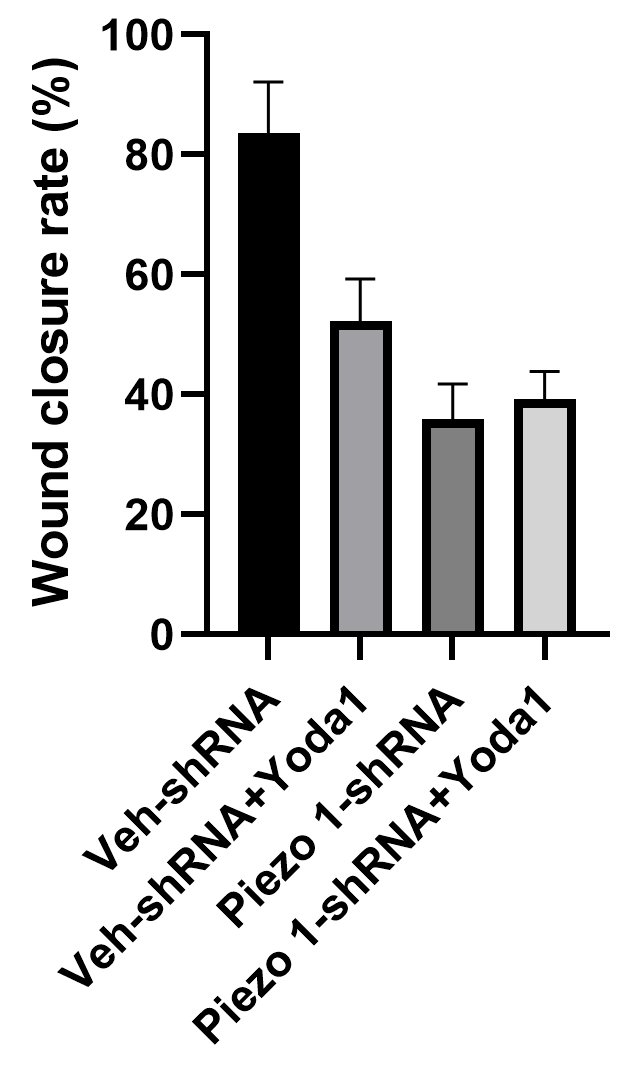


Figure S3. Cell migration in stable knockdown of PIEZO1 A-1847 cells with Yoda1.


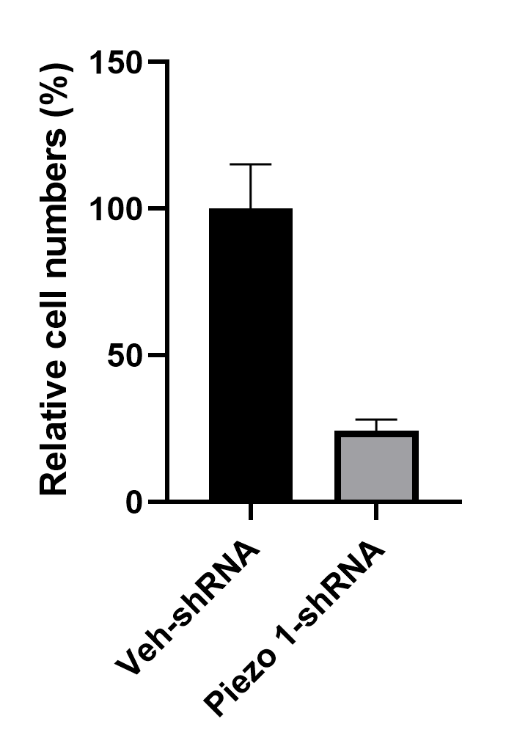


Figure S4. Cell numbers in Piezo1 knockdown A-1847 cells revealed by Transwell assay.
